# Gut microbiota dynamics in free-range layer hens during outbreaks of spotty liver disease and fowl cholera—a field study

**DOI:** 10.1007/s00253-026-13965-0

**Published:** 2026-07-20

**Authors:** Friedrich Petranyi, Maria M. Whitton, Edina Lobo, Sung J. Yu, Ivan Milošević, Dragana Stanley, Yadav S. Bajagai

**Affiliations:** 1 Institute for Future Farming Systems, Central Queensland University, Rockhampton, QLD 4702 Australia; 2 Faculty of Veterinary Medicine, University of Belgrade, Bulevar Oslobodjenja 18, Belgrade, 11000 Serbia

**Keywords:** Microbiota, Layer hen, Spotty liver disease, Precision glycan, Fowl cholera

## Abstract

**Abstract:**

The livestock industry faces growing constraints due to antimicrobial resistance, particularly in poultry systems that have historically relied on antibiotic use. These challenges are exacerbated in free-range production, where restrictions on antimicrobial interventions coincide with heightened exposure to infectious diseases, leading to increased mortality and reduced productivity. On-farm disease outbreaks cannot be reproduced in high ethics standards approved animal houses, where they can be modeled to recognize some patterns of pathogen dynamics. While laboratory settings enable rigorous control and monitoring, they lack the environmental, management, production, pathogen pressures and complexity found on industrial farms and therefore cannot realistically recreate disease progression. Here, we present a field study in a layer flock that experienced three disease outbreaks of spotty liver disease (SLD) and fowl cholera (FC). The treatment group was supplemented with a precision biotic, a synthetic glycan product, while weekly performance measures and responses of microbial populations across major gut sections were observed. The precision biotic supplemented birds showed higher cumulative mortality but also higher egg output. However, as this was an unreplicated field study, these differences should be interpreted as descriptive associations. The intestinal microbial community responded differently to supplementation under concurrent environmental and disease pressures. This study provides large-scale field observations during concurrent outbreaks of SLD and FC, offering real-world insights that may inform future controlled studies of disease dynamics and performance management.

**Key points:**

• *Free-range systems increase disease risk, reducing health and productivity in layer flocks.*

• *Precision biotics were associated with higher egg output and higher cumulative mortality in hens.*

• *Gut microbiota and liver congestion differed between precision biotic supplemented and control groups.*

## Introduction

The livestock industry faces numerous challenges within animal production systems. Among these, particular attention is directed to the development of antimicrobial resistance due to historical and ongoing antibiotic use in layer hens, which places significant metabolic pressures and heightens disease susceptibility. Free-range systems, while meeting consumer demands, introduce additional health challenges in poultry production, including biosecurity risks and skeletal and gut health impairments (Hubert et al. 2019). Spotty liver disease (SLD) and other diseases have emerged as critical concerns (Crawshaw and Young 2003; Van et al. 2016, 2017; Moore et al. 2019). *Campylobacter hepaticus*, the agent that causes SLD, can affect birds during and after the onset of lay. *Campylobacter bilis*, a second *Campylobacter* species, has since been identified as an additional cause of SLD (Van et al. 2023). Clinical findings include sepsis, primarily affecting the liver, leading to necrotizing hepatitis. The consequences could involve a notable increase in mortality and a decline in egg production rates, both by up to 10% (Grimes and Reece 2011). The disease primarily affects birds near the peak of production, posing a significant threat to future performance. The lack of comprehensive epidemiological data has hindered robust analysis of how control and prevention strategies affect SLD incidence (Courtice et al. 2018). Although efforts to develop commercial vaccines are underway, they remain in the early stages of development (Courtice 2020, unpublished conference presentation; Muralidharan et al. 2020). SLD remains a leading cause of mortality from infectious diseases within Australian free-range egg production systems, where birds often have to deal with extreme heat stress (Groves et al. 2024; Wilson et al. 2024).

Another frequent disease affecting laying hens is fowl cholera (FC), caused by *Pasteurella multocida*. It is similar to SLD in causing economic losses through increased mortality and reduced egg production due to sepsis and organ failure (Christensen and Bisgaard 2000). FC has been identified as a significant and recurring problem in free-range farms, with mortality rates up to 55% in broilers (Zhang et al. 2004; Singh et al. 2014). While autogenous vaccines are available, their protection is partial, falling short of ensuring complete immunity during outbreaks (Christensen and Bisgaard 2000). On the other hand, preventative treatments with antibiotics such as chlortetracycline have been successful in controlling FC in layers, ducks and turkeys (Hossain et al. 2018). More recently, Saha et al. (2021) and Sabsabi et al. (2021) reported 91% and 38% of *Pasteurella multocida* strains resistant to tetracycline in outbreaks from laying hen commercial farms, respectively. The deficit in new antimicrobials for poultry use, with the reduced efficacy of previously used drugs due to acquired resistance, has prompted restrictions on antibiotic use to safeguard public health in some jurisdictions (Nolan et al. 2020).

Although there are multiple complexities in how birds cope with these health challenges, the gastrointestinal tract (GIT) and its microbiota are critical components, as they are involved in immune system development, promote nutrient utilization and bone growth and maintain production performance (Dai et al. 2022). The elements influencing microbiota composition can also alter productivity, potentially accounting for the variability in performance observed within a specific flock (Diaz Carrasco et al. 2019). Diet also significantly influences the intestinal microbiome in poultry, as dietary components that evade host digestion and absorption serve as substrates for intestinal bacterial growth (Pan and Yu 2014). However, although antibiotics have been considered an effective and cost-efficient strategy for controlling gut health in some systems, they are not routinely used in laying hens in Australia (Pan and Yu 2014). Therefore, there is a need to investigate new alternatives to support animal production.

Recent advances in biotechnology have allowed the synthesis of precision biotics as a new alternative to support GIT health and sustain animal performance. Glycans, complex carbohydrates, are suitable for developing precision biotics, as they participate in numerous metabolic pathways involved in health and disease responses. They contribute to balanced immunity, prevent pathogenic infection, promote the establishment of a healthy microbiota and are increasingly seen as potential therapeutic targets (Zhou and Cobb 2021). They can contribute to a variety of immune-protective mechanisms through glycan-dependent pathogen binding, as demonstrated by Kafkova et al. (2021).

Precision glycans have been studied as a new alternative for vaccine production against pathogens, as is the case for *Clostridium difficile* (the primary cause of nosocomial diarrhea) since they can play a crucial role in inhibiting pathogen GIT colonization through antigen recognition and the induction of specific antibodies (Broecker et al. 2016). They also play a key role in mucin production, which forms a protective homeostatic barrier between the resident microbiota and the underlying immune cells in the GIT, influencing host-microbiota interactions (Bergstrom and Xia 2013).

The nature of the precision biotic used in this study and described by the manufacturer relies on the selection of glycans targeting microbiome modulation pathways related to nitrogen metabolism and short-chain fatty acid production, beyond any particular microbiota profile, through a mixture of chemically designed glycans that specifically promote a beneficial bacterial DNA expression (Walsh et al. 2021) and significantly improve animal performance during disease outbreak (Bergstrom and Xia 2013; Lobo et al. 2023; Petranyi et al. 2024), making them appealing as an antibiotic alternative for farmers, including the commercial layer industry setup where the present study was conducted.

This multiple-natural-disease-outbreak study examines real disease outbreaks in all their complexity to complement controlled animal disease trials that feature high replication, controlled environmental variables and artificially induced disease; however, successful SLD and FC disease trials are very uncommon, and to our knowledge, there are no studies on natural double or triple outbreaks. An on-farm outbreak field-based study approach can provide valuable insights for farmers and farm managers, especially if performance measures are collected regularly and professionally.

In this study, we evaluated a commercial free-range farm in Queensland, Australia, using an animal house specifically developed for research purposes, with a design that allows physical separation of control and treatment groups while maintaining identical feed, water, management and environmental conditions. This specific layer farm was selected based on its history of SLD and FC outbreaks to evaluate microbiota shifts and the potential effects of precision glycan supplementation under commercial production pressures and during disease outbreaks. Precision glycans have been reported to improve response to disease outbreaks in poultry (Bergstrom and Xia 2013; Lobo et al. 2023; Petranyi et al. 2024) and were explored as a supplement in the present field study. This study aimed to improve our understanding of the mechanisms and outcomes of SLD and FC, and to determine whether and to what level precision glycan supplementation could improve layer hen production performance and reduce antibiotic dependence in a free-range commercial environment.

## Materials and methods

### Animal trial

This study received approval from CQ University’s Animal Ethics Committee, with approval number 0000022879. All handling of animals followed the guidelines outlined in the “Australian Code for the Care and Use of Animals for Scientific Purposes” and adhered to the reporting standards outlined in the Animal Research: Reporting of In Vivo Experiments (ARRIVE). Furthermore, the experimental procedures conducted on a commercial farm adhered to the standards outlined in the 4th edition of the “Code of Practice for the Welfare of Domestic Poultry”, as mandated by the laws governing Australia’s States and Territories.

### Experimental design

The study was conducted under commercial production conditions to capture real-world performance and disease dynamics. Due to logistical and industry constraints, replication at the flock level was not feasible; therefore, each treatment was applied to a single production unit. However, several measures were implemented to minimize bias and improve comparability between groups, including the use of birds from a single source flock, identical housing design and capacity, independent but equivalent feeding systems, and alternating allocation of birds during transfer. In addition, outcome assessments such as gut scoring were performed by a blinded external veterinarian. This design allowed controlled comparison within a commercial setting while maintaining industry relevance. Field-based studies of this scale provide complementary insights to controlled trials and are essential for understanding system-level responses under commercial conditions.

The research was carried out at a free-range layer hens aviary farm located in Southern Downs, Queensland, Australia, focusing on investigating the effects of a glycan intervention in ISA Brown hens, a commonly utilized breed in the Australian egg industry. A single flock of 40,000 pullets from the same rearing farm and shed was employed in the study to ensure uniformity in age and consistent management practices and feeding regimes. To avoid selection bias during transfer from the rearing system to the laying house, every pullet truck trip, composed of a maximum load of 7390 pullets, alternated the unloading between both sides of the production shed. The production shed was designed to separate the two flocks transferred from rearing to the point of lay into two groups, each consisting of 20,000 birds. These groups were assigned to two separate sections of the shed, with physical separation ensured by an egg collection room inside the shed and a wired fence in the range area. Each side of the shed was equipped with independent silos and feed lines. The design and capacity of both sides were identical, allowing one group to receive the glycan precision biotic treatment (PB), while the other remained untreated (CT).

The specific glycan used in this investigation was Symphiome™ (DSM-Firmenich, Singapore, Singapore), incorporated into the feed at a dose of 900 g per ton, as recommended by the manufacturer and consistent with previous studies (Blokker et al. 2022; Lobo et al. 2023; Yan et al. 2023; Petranyi et al. 2024). Multiple performance measures were taken daily and weekly. Additionally, in predetermined intervals, a total of 40 birds, 20 from each group, were euthanised via cervical dislocation. Gut scoring, following the methodology outlined by De Gussem (2010), was conducted by an external, contracted, qualified poultry veterinarian who was not associated with the study and was blinded to the birds’ shed allocations. Intestinal and liver samples were collected for 16S amplicon sequencing and histological analysis. Tissue and microbiota sampling occurred at 50 and 72 weeks of flock age, with studies conducted on ileum content and mucosa-associated microbiota, caecal and gizzard content microbiota, and microbiota from 100 cloacal swabs collected from each treatment group at each time point. Additionally, samples were collected for histopathological examination of the ileum and liver.

### Performance and productivity parameters

The commercial farm where the study was conducted has a long history of research and development, with meticulous performance monitoring using modern equipment and methods. Several performance measures were collected daily by farm staff and the team of veterinarians to assess poultry production and egg quality across all operational poultry sheds. These measures included mortality, encompassing daily and cumulative counts of deceased birds, and the rate of lay (ROL), calculated as the percentage of eggs produced daily, relative to the number of birds in the shed. Cumulative hen-housed eggs were also recorded, and the total number of eggs laid was compared to the initial number of birds placed at the point of lay.

The grams of feed consumed per bird per day (GBD) were also monitored. It was measured using weight cells under the shed’s silos to determine the total feed used per day on each side of the shed. Feed conversion was assessed, measuring the kilograms of feed required to produce one dozen eggs and one kilogram of eggs. Bird body weight was measured weekly at various locations in the shed to obtain a representative sample, with 100 birds randomly weighed per group.

The cumulative number of dirty eggs excluded from the initial grading due to unsuitability for consumption was also recorded. Egg weight was routinely measured, along with eggshell thickness, which was assessed in grams and millimeters, respectively. Internal egg quality, measured as Haugh units, was evaluated to assess egg protein quality by correlating the height of the egg white albumen with total egg weight. Additionally, the average yolk color was evaluated on a scale of 1 to 16 using the DSM yolk color fan (DSM, Singapore, Singapore, version 2020) as a reference. The egg quality parameters previously listed were analyzed from a sample of eight eggs per testing group, collected on the same day each week. By the end of the trial, 944 eggs were tested. The testing was conducted using a Digital Egg Tester, Model DET6500 (Nabel Co. Ltd., Kyoto, Japan.). These performance measures serve as critical indicators for evaluating various aspects of poultry production and egg quality.

Considering the large scale of the trial, the occurrence of naturally occurring disease outbreaks, and the inability to set up group replicates, the performance results were used only for descriptive purposes, with no statistical analysis.

### DNA extraction and sequencing

The DNA extraction procedure followed a cell lysis protocol tailored for 16S microbiota analysis as outlined by Yu and Morrison (2004). Subsequently, the lysate was purified using a DNA spin purification kit (Enzymax LLC, Cat# EZC101, Lexington, USA). The quality and quantity of DNA were evaluated using a NanoDrop spectrophotometer (Thermo Fisher Scientific, Waltham, MA, USA). For PCR amplification, a custom barcoding system with primers Pro341F and Pro805R (Takahashi et al. 2014) was utilized in conjunction with New England Biolabs Q5 High-Fidelity 2X PCR Master Mix (Ipswich, MA, USA).

After DNA extraction and PCR amplification, the amplicon library was purified using the AMPure XP Bead-Based Reagent (Beckman Coulter, Lane Cove, Australia) according to the manufacturer’s protocol. The sequencing was performed on the Illumina MiSeq platform (Illumina, San Diego, CA, USA) using a 2 × 250 bp paired-end configuration.

### Histological analysis

The ileum and liver tissue samples were collected and preserved by immersion in a 10% buffered formalin solution. Subsequent processes, including tissue processing, paraffin embedding, sectioning and staining, were outsourced to the University of Queensland (Gatton, QLD, Australia).

The ileum slides underwent staining using the Periodic Acid-Schiff-Alcian Blue staining method (Yamabayashi 1987), while the liver slides were stained with hematoxylin and eosin (H&E) stain (Fischer et al. 2008). All slides were digitally scanned using Panoptiq™ software from ViewsIQ Inc. (Vancouver, BC, Canada) and a Nikon Eclipse Ci-L Plus biological microscope (Nikon, Tokyo, Japan).

### Statistical analysis

#### Bioinformatics

Data analysis was conducted using the Quantitative Insights into Microbial Ecology 2 (QIIME 2) software package (Bolyen et al. 2019). Sequencing errors were rectified, and chimeric sequences were removed from the dataset using the DADA2 plugin with recommended settings (Callahan et al. 2016). Preliminary taxonomy assignments were performed utilizing the SILVA database (Quast et al. 2013). Outputs from the QIIME analysis were further analyzed using various R (version 4.4.2) software packages, including phyloseq v1.50.0 (McMurdie and Holmes 2013), microecco v1.15.0 (Liu et al. 2021), vegan 2.7-1 (Oksanen et al. 2016) and microbiome v1.28.0 (Lahti and Shetty 2018). To account for uneven library sizes, sequence counts were rarefied prior to downstream analysis.

#### Histology

Essential morphometric parameters for the ileum included villus height (the distance from the tip to the base of the villi), villus width (the mean value between the basal and apical villus width) and crypt depth (the distance from the crypt neck to its base). Additionally, goblet cells in both villi and crypts were enumerated. These parameters were assessed using 10 slides per group, with 10 villi per slide, and analyzed with GraphPad Prism 9 (GraphPad Software, San Diego, CA, USA) using a nonparametric Mann–Whitney *U* test. To determine the villus surface area, the previously proposed equation (Rubio et al. 2010) was employed: Villus surface area [*μ*m^2] = π × Villus height [*μ*m] × Villus width [*μ*m]. This equation enables estimation of surface area from villus dimensions. Liver congestion was assessed semi-quantitatively and scored as 0 (absent), 1 (mild), 2 (moderate) and 3 (severe).

#### Gut scoring

All birds chosen randomly for necropsy and gut sampling at each sampling point were also utilized for gut scoring analysis. Scoring was conducted by experienced veterinarians who were blinded to bird groups to remove bias. This analysis employed the “Macroscopic Scoring System for Bacterial Enteritis in Broiler Chickens and Turkeys” (De Gussem 2010). The system assesses various factors, including gut ballooning, dilated blood vessels, wall thickness, inversion reflex, abnormal content and undigested feed.

## Results

### Animal health and performance

**Fig. 1:**
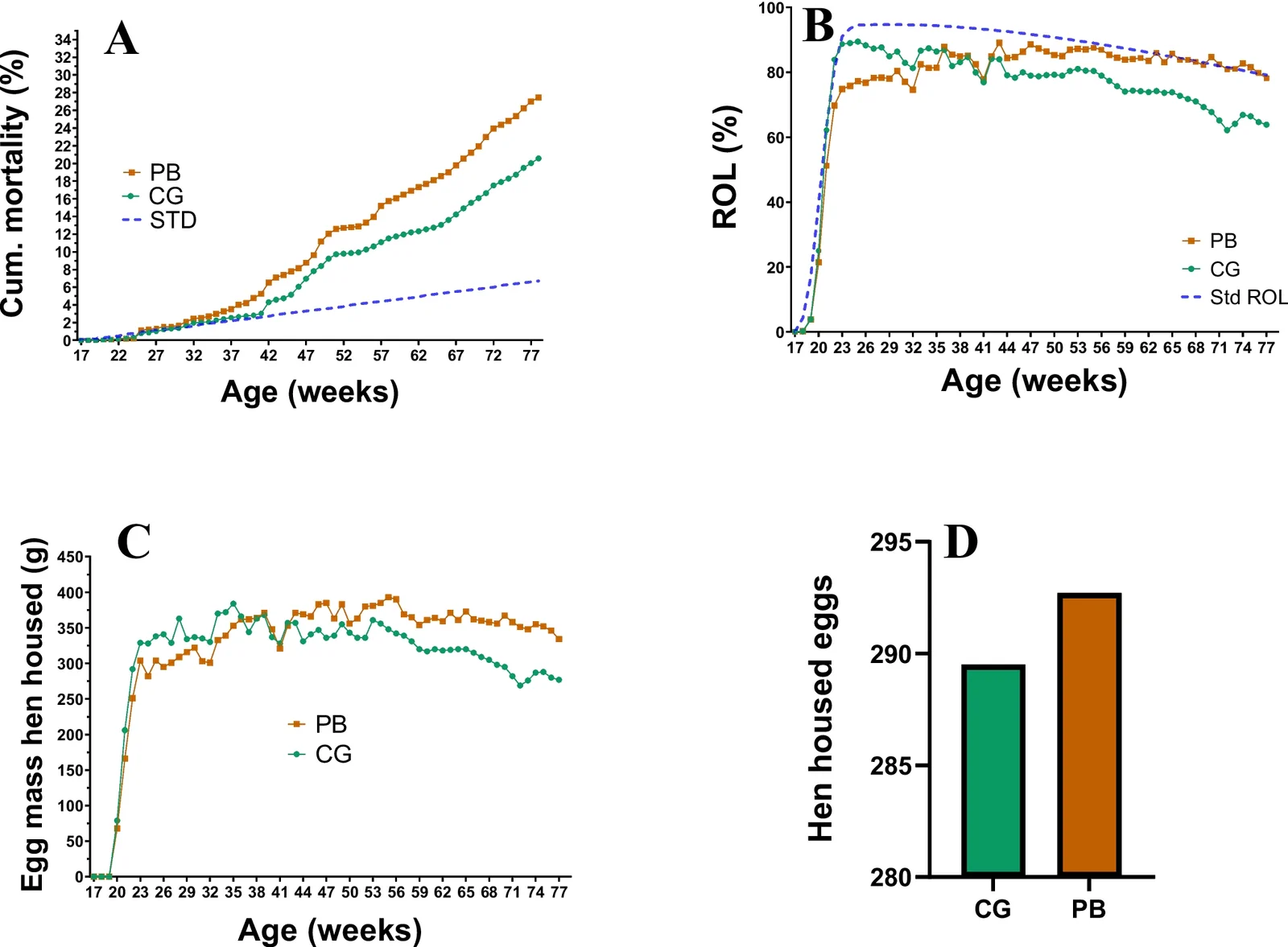
**A** Cumulative mortality, **B** rate of lay (ROL), **C** weekly egg mass per hen housed, and **D** total hen housed eggs from placement at the point of lay at 17 weeks until the end of the trial at 77 weeks of age. The blue dotted line represents the breed standard (CT control, PB precision biotic)

At the end of the production cycle, at 77 weeks of age, cumulative mortality exceeded the breed standard in both groups, primarily due to two outbreaks of SLD at 40 and 50 weeks of age and an outbreak of FC at 70 weeks of age. The final cumulative mortality was numerically higher in the precision biotic-supplemented (PB) group than in the control (CT) group, at 27.44% and 20.56%, respectively (Fig. 1A). The outbreaks were identified by the farm veterinarian through necropsy, isolation and PCR-based pathogen identification from liver samples.

The rate of lay was higher in PB than in CT across the overall trial, especially from 36 weeks of age, and performance returned to the breed standard from week 65 of age (Fig. 1B).

At the end of the trial, feed efficiency was better in CT than in PB: 1.69 versus 1.91 kg of feed/dozen and 2.37 versus 2.70 kg of feed/kg of eggs, respectively. The cumulative eggs per hen housed were higher in PB, delivering 3.75 extra eggs per bird at the end of the trial. Similar differences were observed in cumulative egg mass: PB 17.68 kg and CT 17.38 kg per hen housed. This was also reflected in higher weekly egg mass per hen housed in PB than in CT from week 37 of age through the end of the trial (Fig. 1). No notable differences were observed in egg quality parameters, including egg weight, break strength, Haugh units, yolk colour or shell thickness, after analysing 944 eggs over the 59-week laying period, even during the three major disease outbreaks described.

No significant differences were observed in dysbiosis score, gut integrity or abnormal content during gut scoring at either mid- or late-lay.

### Mid-lay microbiota

**Fig. 2:**
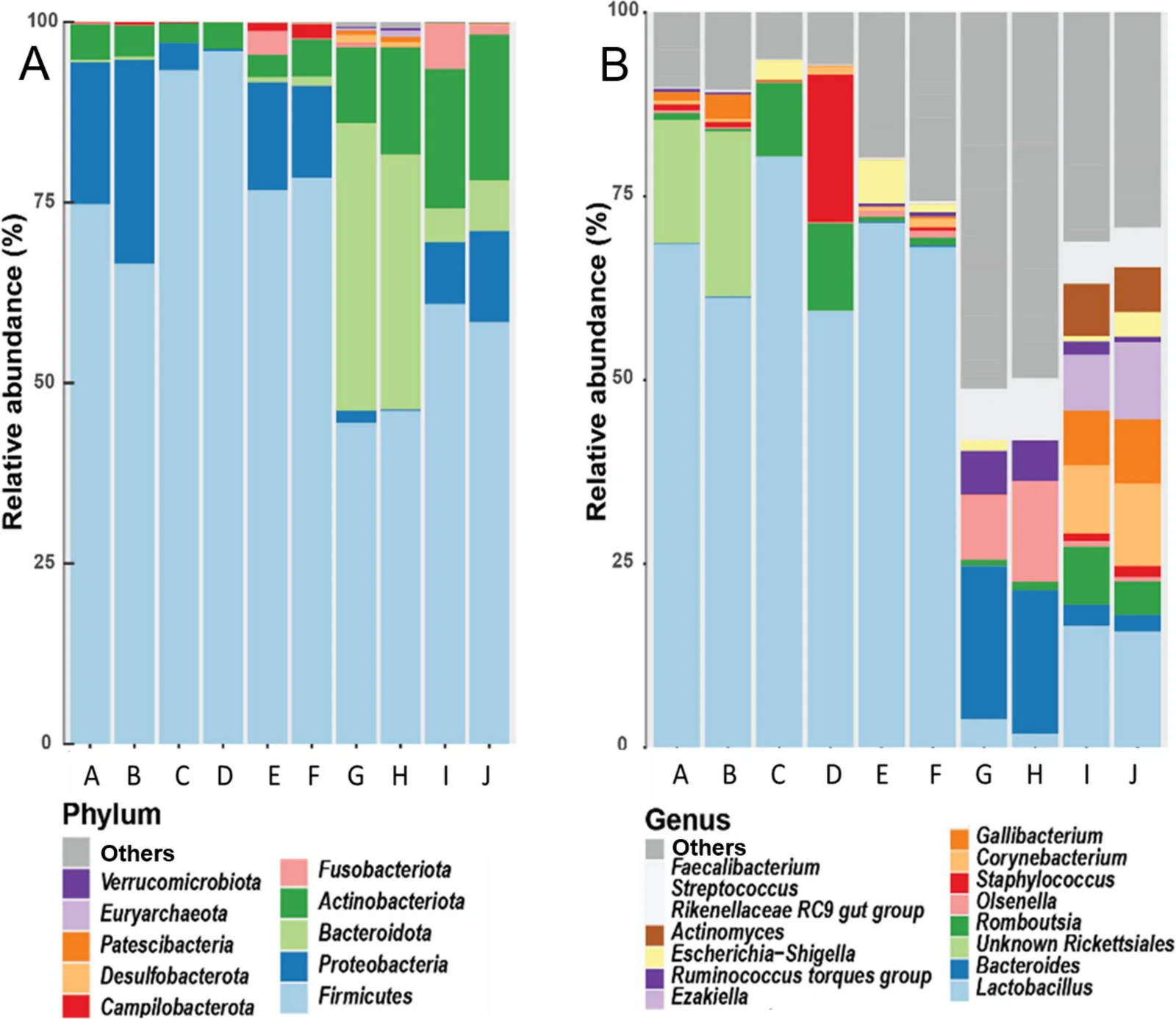
Microbiota analysis showing the top ten most abundant phyla (panel **A**) and the top 15 genera (panel **B**) present during mild-lay at 50 weeks of age: **A** gizzard CT, **B** gizzard PB, **C** ileum content CT, **D** ileum content PB, **E** ileum mucosa CT, **F** ileum mucosa PB, **G** cecum CT, **H** cecum PB, **I** cloacal swabs CT, **J** cloacal swabs PB

During the mid-lay period, community membership showed similarities between groups and was predominantly composed of the *Firmicutes* phylum across all sampled sections. Notably, in the cecum, *Bacteroidota* exhibited a prevalence similar to that of *Firmicutes* in both the CT and PB groups (Fig. 2). At the genus level, *Lactobacillus* dominated in both groups across samples from the gizzard, ileal contents, ileal mucosa and cloacal swabs. Similarly, at the phylum level, the cecum showed a distinctive abundance compared to other sections, with both groups showing high abundance by the *Bacteroides* genus.

In the gizzard, Unknown *Rickettsiales* was among the dominant taxa in both groups, but in the ileum, PB showed *Staphylococcus* and *Romboutsia* as the prevalent taxa.

In the ileum mucosa, CT showed secondary dominance of *Escherichia-Shigella*, whereas PB showed a more diverse composition. Both groups exhibited similar cecal microbiota compositions, with *Olsenella* emerging as the second-most abundant genus after *Bacteroides*. Similarly, both groups shared a similar microbiota profile in the cloacal swab, with *Corynebacterium* as the second-most abundant genus.

**Fig. 3:**
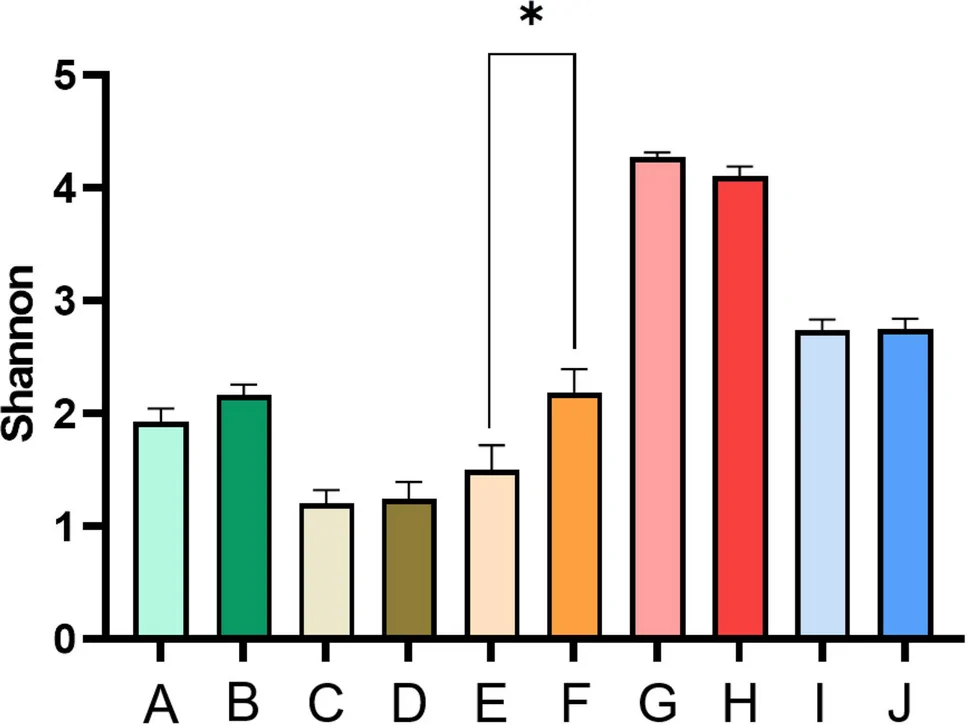
At 50 weeks of age, the Shannon diversity index shows a significant increase in PB ileum mucosa (Mann–Whitney false discovery rate (FDR)-corrected *p = 0.03000, CT control, PB precision biotic). **A** Gizzard CT, **B** gizzard PB, **C** ileum content CT, **D** ileum content PB, **E** ileum mucosa CT, **F** ileum mucosa PB, **G** cecum CT, **H** cecum PB, **I** cloacal swabs CT, **J** cloacal swabs PB

Analysis of alpha diversity across sections revealed a trend in which the Shannon index increased from the upper to lower gastrointestinal tract sections, particularly at the cecum and cloaca. However, no consistent differences in alpha diversity between the groups were observed, except in the ileum mucosa, where PB showed higher alpha diversity (Mann–Whitney, false discovery rate (FDR)-corrected *p* = 0.0300; see Fig. 3). The beta-diversity analysis revealed that microbial communities from both groups clustered by intestinal section, with low dispersion during the mid-lay period.

**Fig. 4:**
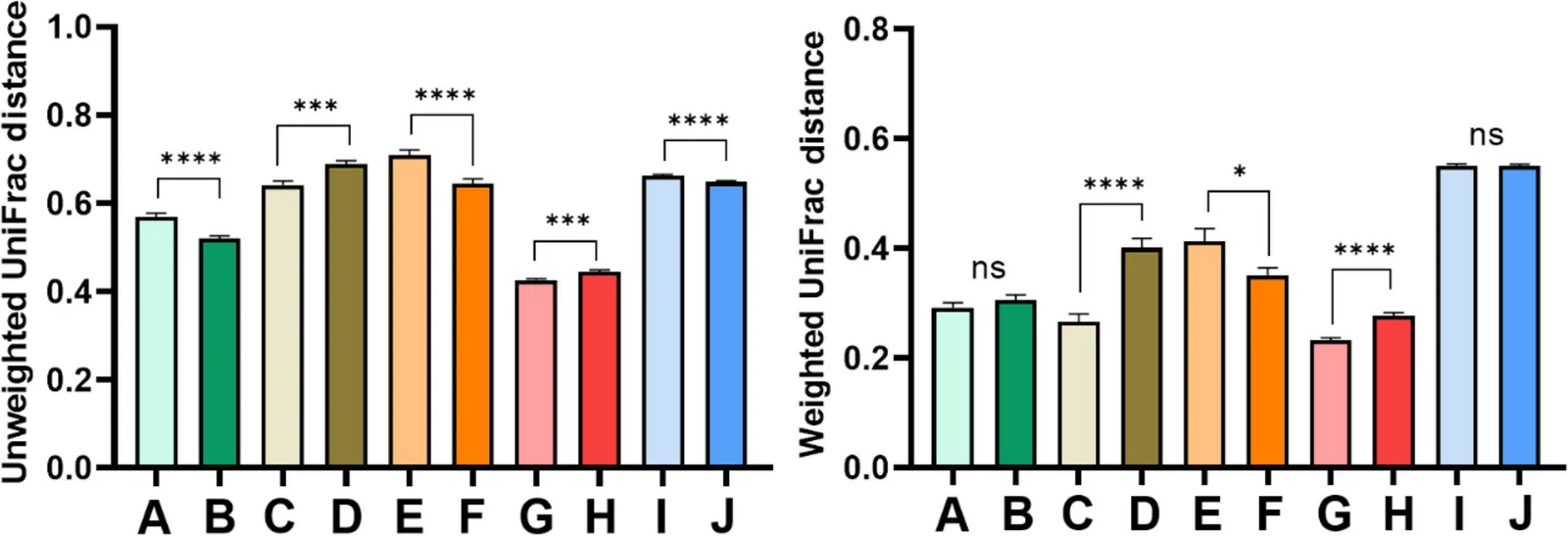
Unweighted and weighted UniFrac distances within each group (CT, control, and PB, precision biotic) during the mid-lay production phase at 50 weeks of age. Significance is indicated with asterisks (* = *p* < 0.05; **** = *p* < 0.0001; ns = not significant) and calculated using FDR corrected Mann–Whitney test: **A** gizzard CT, **B** gizzard PB, **C** ileum content CT, **D** ileum content PB, **E** ileum mucosa CT, **F** ileum mucosa PB, **G** cecum CT, **H** cecum PB, **I** cloacal swabs CT, **J** cloacal swabs PB

Significant dissimilarities in microbial communities between the groups were observed across all sections, as indicated by Unweighted UniFrac distances (*p* < 0.0001 to *p* < 0.001, Fig. 4). However, when relative abundance was incorporated into the analysis, these differences were not significant in the gizzard and cloacal swabs (Weighted UniFrac).

**Fig. 5:**
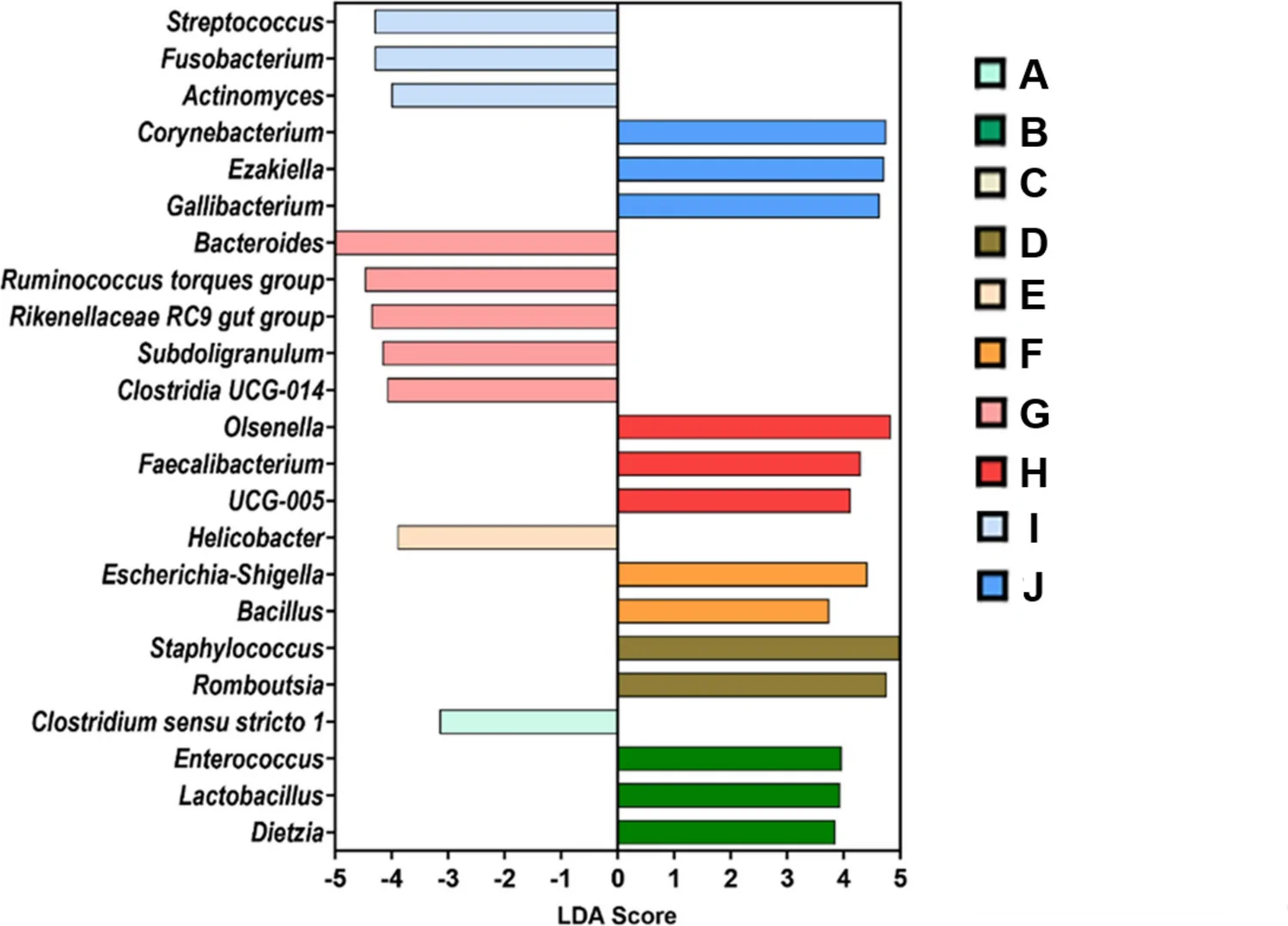
LEfSe analysis at 50 weeks of age (Mid-lay) (LDA: linear discriminatory analysis). **A** Gizzard CT, **B** gizzard PB, **C** ileum content CT, **D** ileum content PB, **E** ileum mucosa CT, **F** ileum mucosa PB, **G** cecum CT, **H **cecum PB, **I** cloacal swabs CT, **J** cloacal swabs PB. CT, control and PB, precision biotic

The linear discriminant analysis identified specific genus markers across different sections and between groups, revealing a predominant composition of fermentative and lactic acid-producing genera. Some of these findings are particularly relevant to the poultry industry. Notably, in the PB group, potential pathogens, such as *Escherichia-Shigella* and *Gallibacterium*, were identified as marker genera in ileum content and cloacal swabs, respectively. In contrast, *Lactobacillus*, a genus commonly associated with probiotic bacteria, was highlighted as a PB marker in the gizzard. In the CT group, *Helicobacter* was identified as a primary marker in ileal contents (Fig. 5).

### Late-lay microbiota

**Fig. 6:**
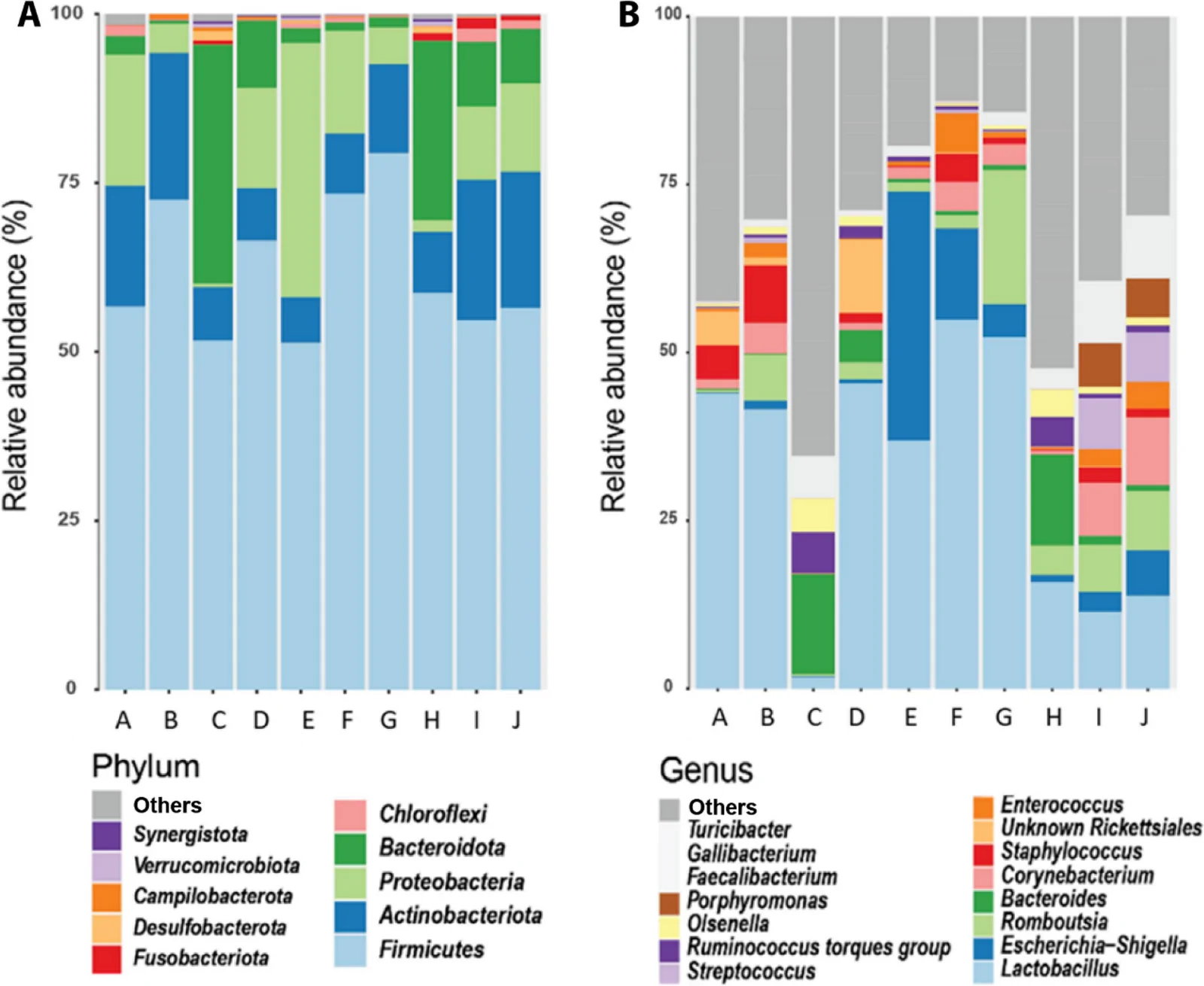
Top ten most abundant phyla and top 15 genera in late-lay microbiota at 72 weeks of age: **A** gizzard CT, **B** gizzard PB, **C** ileum content CT, **D** ileum content PB, **E** ileum mucosa CT, **F** ileum mucosa PB, **G** cecum CT, **H** cecum PB, **I** cloacal swabs CT, **J** cloacal swabs PB

During the late-lay period, community membership showed similarities among groups and was predominantly composed of the *Firmicutes* phylum across all sampled sections. Following *Firmicutes*, *Proteobacteria*, *Actinobacteriota* and *Bacteroidota* were the abundant phyla across different sections in both groups (Fig. 6).

At the genus level, *Lactobacillus* dominated both groups across samples in the gizzard, ileum content, ileum mucosa and cloacal swabs, except in the CT ileum content (Fig. 6, panel B, column C), where *Bacteroides* was more abundant, with low *Lactobacillus* abundance.

**Fig. 7:**
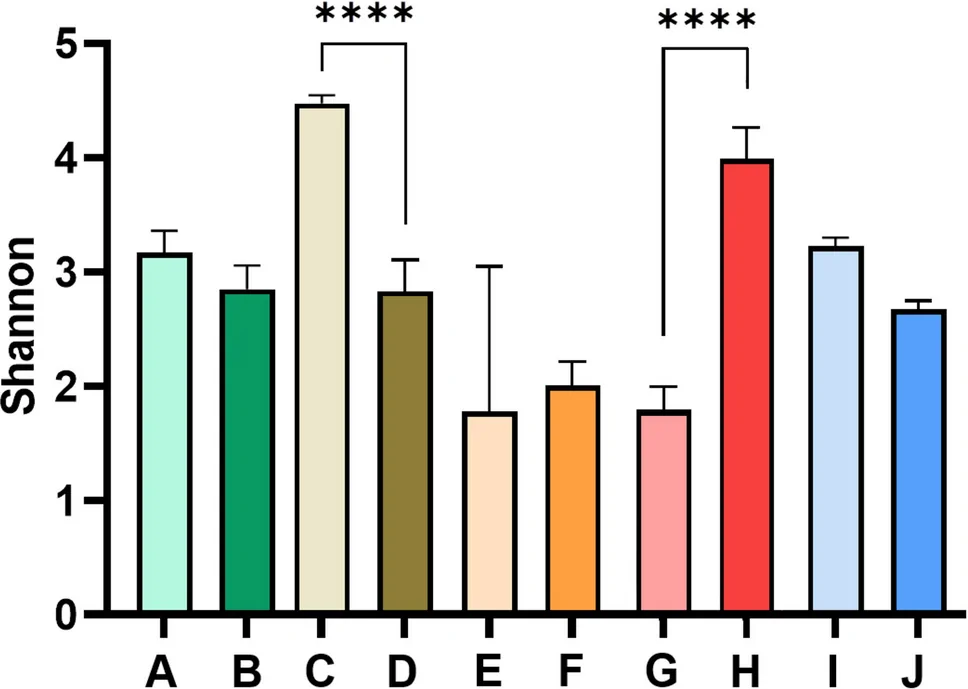
Alpha diversity during late-lay at 72 weeks of age (**** = *p* < 0.00001) (CT control, PB precision biotic). **A** Gizzard CT, **B** gizzard PB, **C** ileum content CT, **D** ileum content PB, **E** ileum mucosa CT, **F** ileum mucosa PB, **G** cecum CT, **H** cecum PB, **I** cloacal swabs CT, **J** cloacal swabs PB

The Shannon alpha diversity across sections revealed significant differences between groups. The CT group exhibited higher microbiota diversity in ileal contents (Fig. 7). In comparison, PB showed greater cecal diversity, in contrast to the unusually low diversity observed in CT for this GIT section (Mann–Whitney FDR-corrected *p* < 0.00001; see Fig. 7). No significant differences were observed in the remaining sections. Similar to the mid-lay period at 50 weeks of age, the beta diversity analysis showed that samples from both groups clustered by gut section rather than by treatment.

**Fig. 8:**
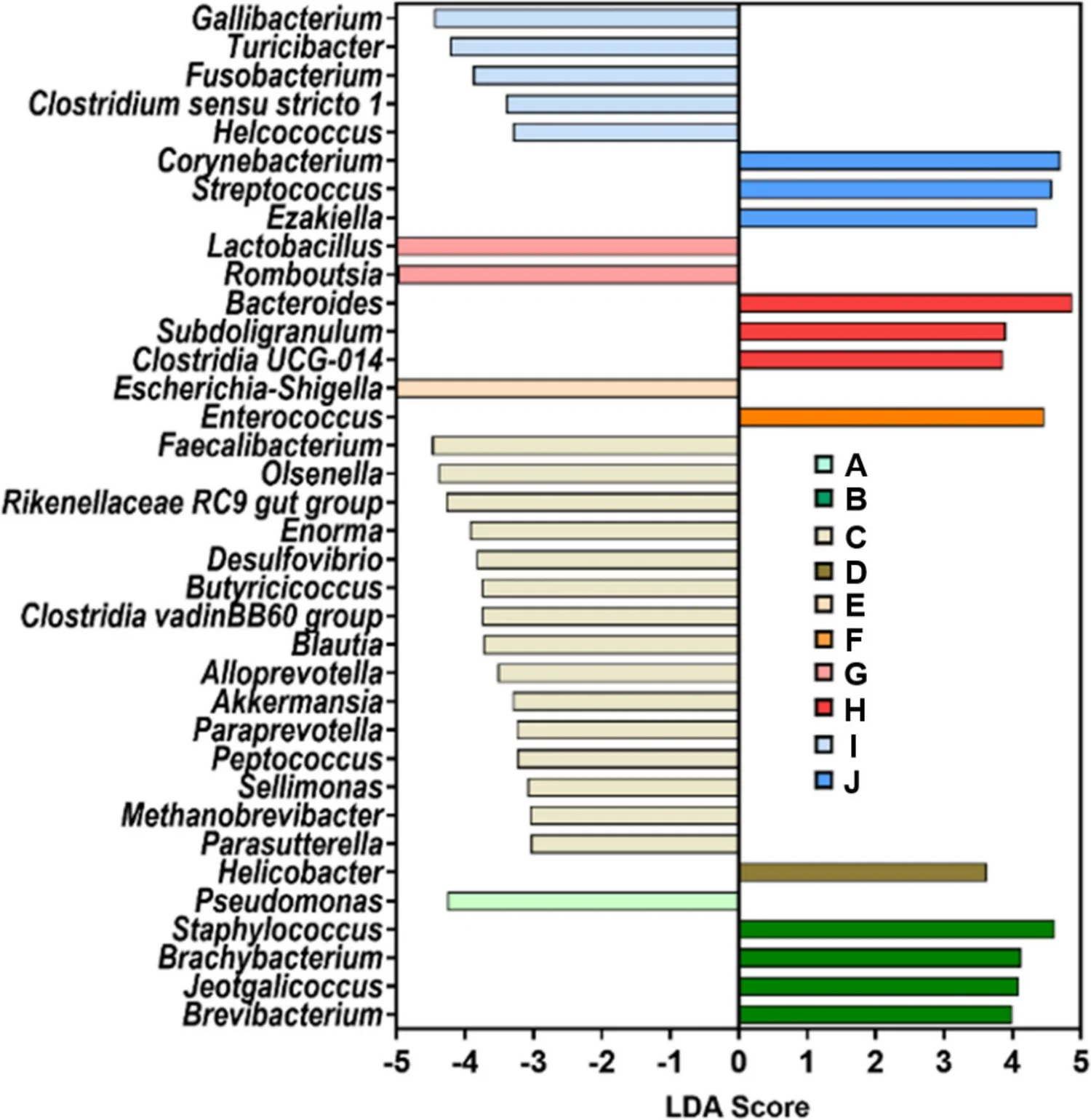
LEfSe analysis at 72 weeks of age (LDA: linear discriminatory analysis) (CT control, PB precision biotic). **A** Gizzard CT, **B** gizzard PB, **C** ileum content CT, **D** ileum content PB, **E** ileum mucosa CT, **F** ileum mucosa PB, **G** cecum CT, **H** cecum PB, **I** cloacal swabs CT, **J** cloacal swabs PB

During the late-lay period, linear discriminant analysis identified specific genus markers across sections and between groups. Some of these findings are of notable significance to the poultry industry. Specifically, in the CT group, *Lactobacillus*—a genus commonly associated with probiotic bacteria—was more abundant in the cecum, and *Escherichia-Shigella*, a potential pathogen genus, was identified in the CT ileal mucosa. In contrast, *Staphylococcus* in the gizzard was a marker in the PB group (Fig. 8).

Significant dissimilarities were observed in the microbial communities between CT and PB in ileum content, cecum and cloacal swab samples, as indicated by Unweighted UniFrac, with significant values (*p* < 0.00010 to *p* < 0.01000). However, when incorporating relative abundance using Weighted Unifrac, only ileum content and cloacal swabs retained significant differences.

### Histological analysis

**Fig. 9:**
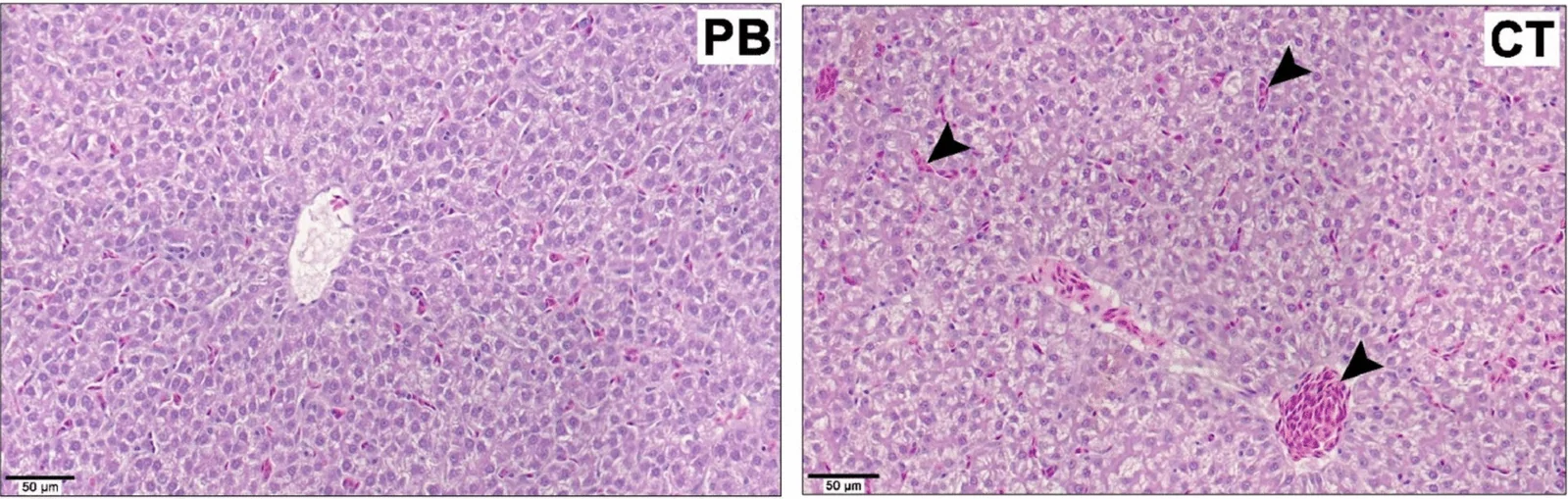
Representative photomicrographs of liver tissue sections from control (CT) and precision biotic (PB) groups (*p* < 0.02000). Arrowheads mark congested sinusoids (bar: 50 µm) (haematoxylin and eosin staining method)

Histological examination of liver tissue revealed preserved tissue architecture and no pathological alterations in both groups. The only distinction observed was that livers from the control group displayed markedly higher congestion scores (*p* < 0.05, Fig. 9). No significant differences between groups or ages were observed in ileum goblet cell abundance or histomorphology at the villus or crypt level.

## Discussion

Free-range farming environments present unique challenges for animal production (Campbell et al. 2021), necessitating a delicate balance in maintaining physiological equilibrium, particularly at the gastrointestinal level, due to heightened exposure to various pathogens (Jeni et al. 2021). As in this study, birds in free-range environments may be more likely to develop bacterial infections, such as SLD. Furthermore, egg production can be significantly affected by birds spending more time in the range than by birds staying indoors throughout the laying cycle (Sibanda et al. 2020). The interplay between host health and environmental factors becomes increasingly pronounced, as evidenced by the complexities observed in both groups under study.

Throughout the trial period, disease outbreaks, including two SLD outbreaks and one FC outbreak, accentuated the vulnerability of poultry in free-range settings. These pathogens, known for their detrimental effects on layer hens, necessitated antibiotic treatments in both experimental groups. Despite efforts to mitigate these challenges, PB had a larger negative impact on cumulative mortality and feed conversion. Even though these acute outbreaks notably affected both groups relative to the breed standard, PB had a higher egg output metric, consistent with our previous findings (Petranyi et al. 2024).

Higher mortality in PB does not align with previous research by Lobo et al. (2023), who reported in a commercial on-farm study with replicated groups that precision glycans significantly reduced mortality during a naturally occurring Infectious Bronchitis outbreak. Additionally, in a previous study conducted by our research team on the same farm where the present case study was carried out, mortality was lower during high-mortality smothering events when using precision glycans (Petranyi et al. 2024). However, smothering is a complex behavioral phenomenon, which is not well understood and is likely influenced by multiple factors (Bright and Johnson 2011; Gebhardt-Henrich and Stratmann 2016), and thus may not be reproducible across settings.

Several explanations may account for the seemingly paradoxical higher mortality, yet higher egg output metric in the PB group. It is possible that PB stimulates hens to lay beyond their physical limits, thereby inducing excessive metabolic stress. A possible scenario is that PB can increase mortality, potentially reducing the proportion of low-performing or compromised birds and thereby increasing production among the remaining flock. This would then resemble the common process of removing non-laying birds via the process of culling, which reduces total flock maintenance costs and allows healthier birds access to more resources. These interpretations remain speculative and require further investigation and validation.

Transitioning to late-lay, the *Firmicutes* phylum predominated in both groups, but its relative abundance decreased compared to mid-lay. This suggests the influence of environmental factors and microbiome maturation (Joat et al. 2021) on the establishment of microbial ecology. It was previously reported (Dai et al. 2022; Joat et al. 2023; Bajagai et al. 2024) that the relative abundance at the phylum level initially favored *Firmicutes* and, with age, shifted to a predominance of *Bacteroidetes*. However, this was detected only in the ileal content on CT and in the cecum on PB, and was not observed in the other sections under study, with *Firmicutes* remaining the dominant phylum, although *Actinobacteria* and *Proteobacteria* showed marked increases.

The mid-lay LEfSe analysis, which shows *Faecalibacterium* as a specific marker in the cecum at PB, is also worth noting, as this beneficial genus produces high levels of butyrate in the gastrointestinal tract (Rowland et al. 2018). These metabolic adaptations may have contributed to the observed differences in egg production and, in theory, support the presumed role of glycan utilization under adverse conditions during the laying hen production cycle. However, a replicate study should be performed to confirm it.

The LEfSe analysis contributed additional insights. The increase of *Enterococcus* and *Lactobacillus* in the gizzard of the PB group highlighted an intricate balance between commensal, probiotic and potentially pathogenic species during mid-lay. Conversely, CT showed *Clostridium *sensu stricto 1 as the main marker in the gizzard during the same period; however, while this genus includes *Clostridium perfringens*, it is dominated mainly by beneficial or non-pathogenic species (Gupta and Gao 2009; Li et al. 2023). It must be noted that the investigation of microbial communities using 16S-based methods can reliably identify changes in microbial structure and diversity, but cannot identify taxa at the species level or distinguish neutral, beneficial and pathogenic strains within a genus. Thus, we can reliably claim only that the present findings support that PB is associated with shifts in microbial diversity, composition and community structure, rather than specific functional or pathogenic outcomes.

In parallel with the observed microbiological shifts, the macroscopic assessment of gut integrity, as indicated by the dysbiosis score, revealed no significant differences between groups, despite pronounced variations in microbial communities and reported performance metrics. It is worth noting, however, that the overall dysbiosis scores in both groups remained low, averaging 1.20 at 50 weeks and 1.60 at 72 weeks of age, on a scale where 10.00 indicates the most compromised gut health. However, a closer histological examination revealed considerable disparities. Specifically, histological analysis of liver tissue sections from the CT group showed significantly increased congestion of hepatic blood vessels, a feature commonly associated with liver pathology (Zaefarian et al. 2019). This finding underscores the complexity of liver health, as previous studies have demonstrated that probiotic and prebiotic supplementation can modulate hepatic sinusoid congestion in poultry (Pithva et al. 2015; Abd El-Ghany et al. 2022).

Recent research has highlighted the potential of precision glycans to alleviate liver congestion in poultry, suggesting a beneficial role in hepatic health (Lobo et al. 2023). However, the improvement in liver health is likely attributable to changes in the dynamics of SCFA (short chian fatty acid)-producing bacteria, potentially influenced by dietary precision glycans. Improved liver health during harsh environmental conditions, combined with disease outbreaks, may indicate the activation of previously undetected beneficial pathways that could support bird performance in a highly disease-challenged environment.

This study has some limitations inherent to the field design. The trial was conducted using a single production unit per treatment, and therefore, the flock represents the experimental unit. As a result, the observed differences in mortality, production performance and intestinal microbiota should be interpreted as associations under commercial conditions rather than causal effects. In addition, the study was conducted during concurrent outbreaks of SLD and FC, introducing substantial biological and environmental complexity that may have influenced both performance and microbiome outcomes. While such conditions limit experimental control and increase the potential for confounding, they also reflect real-world production systems where multiple stressors and disease challenges occur simultaneously. Accordingly, these findings provide ecologically relevant insights that may inform hypothesis generation and guide the design of future controlled and replicated studies. Future replicated studies that incorporate detailed cost data would also enable a formal economic evaluation, weighing the higher egg output of supplemented birds against their higher mortality and poorer feed conversion.

## Conclusions

Free-range commercial farming environments expose poultry to increased pathogen loads, posing challenges to health and productivity. This field study documented disease outbreaks, including SLD and FC, in a commercial layer flock, and described the associations in performance, gut microbiota and liver histology between a precision biotic supplemented group and an untreated control group. Microbiota compositional differences were observed between the two groups across multiple gut sections, and lower hepatic congestion was recorded on the supplemented side, but these observations require confirmation in replicated, controlled trials. On-farm field studies of disease outbreaks are critical for improving our understanding of epidemiological and clinical information, enabling veterinarians to differentiate between random and recurrent persistent issues, thereby guiding precise and cost-effective intervention and, in this way, protecting both industry and public health. By increasing the available on-farm outbreak data, veterinarians can gain a better understanding and second-hand experience, predict potential issues, optimize and adjust biosecurity, and frame rapid control measures. An abundance of on-farm disease outbreak data can help understand environmental complexity, management differences and strategies, pathogen strain variability and the influence of different bird genetics. The information from such studies can inform the experimental design of animal house studies, which are crucial for testing treatments and vaccines and for understanding infection mechanisms, virulence factors and immune responses that cannot be fully determined through real-time on-farm disease outbreaks.

## Data Availability

Data associated with this study have been deposited in the NCBI SRA database under the accession number PRJNA1034760.

## Declarations

### Use of generative AI tools and language models statement

The authors confirm the use of the following software: Grammarly (Version 2025 PRO), which was used to assist with the grammar and spelling of the primary document. The authors declare that no AI tools were used to generate content or analyse the results of this study, and that the research team produced the entire study.
